# Supplementary material for: Multilevel factors associated with delays in screening, diagnosis, and treatment for lung cancer—A mixed methods systematic review protocol
Source: PLoS One. 2024 Oct 11;19(10):e0309196. doi: 10.1371/journal.pone.0309196 (PMC11469495; doi:10.1371/journal.pone.0309196)
Supplement: Attachment — Submitted filename: Response to Reviewers_7.29.2024.docx [file pone.0309196.s004.docx]

**Response to Reviewers’ Comments**

Additional Editor Comments:

I believe you have addressed the comments from the reviewers but ask that you review your use of the word fatal in the sentence, "For quantitative studies, a lack of control for confounding will be considered a fatal flaw” and rewrite this sentence.

**Response:** We have deleted the sentence, as given the comment, deletion does not detract from the methodology.

Reviewer #3: Thank you for the opportunity to review this manuscript. The authors have considerably responded to the reviewer’s comments. However, I would like to make some minor suggestions to improve the manuscript.

**Response:** We have addressed the comments. See below.

Objectives
Page 6, lines 93…The authors should go through their objective to correct the sentence or add (is) to “the objective of this systematic review (is) to identify multilevel factors associated with delays experienced by patients along the lung cancer care continuum from screening to diagnosis and treatment in the US”

**Response:** We have applied the suggestion.

Outcomes
Page 6, line 110…” Delay is defined based…” (be) should be removed from the sentence.

**Response:** We have applied the suggestion

Eligibility criteria: page 9, lines 132-133. What happens where both reviewers do not reach a consensus?. Will the authors involve a third reviewer to resolve their differences or how do they intend to resolve their discrepancies when the both of them disagree.

**Response:** To address this comment, we added the following sentence: “In the event that consensus is not reached, input will be sought from a third reviewer to resolve the discrepancy.”
